# Stable H3 peptide was delivered by gold nanorods to inhibit LSD1 activation and induce human mesenchymal stem cells differentiation

**DOI:** 10.18632/oncotarget.15487

**Published:** 2017-02-18

**Authors:** Xin Meng, Jianping Li, Minjuan Zheng, Lei Zuo, Chao Sun, Yongsheng Zhu, Ling Fang, Liwen Liu, Xiaodong Zhou

**Affiliations:** ^1^ Department of Ultrasonography, Xijing Hospital, Fourth Military Medical University, Xi'an 710032, Shaanxi, China; ^2^ Department of Radiation Oncology, Xijing Hospital, Fourth Military Medical University, Xi'an 710032, Shaanxi, China

**Keywords:** gold nanorods (AuNRs), hepatosis, stable peptide, LSD1, human mesenchymal stem cell (human MSCs)

## Abstract

Recently, lysine-specific demethylase 1 (LSD1), which is the first identified histone demethylase, regulates post-translational modifications and has great promise as new targets for cancer and other diseases. Moreover, the ability of LSD1 to induce the differentiation of stem cells has attracted great attention in biological fields. In this study, we designed LSD1 peptide inhibitor based on its substrate H3 peptide. Through introducing a disulfide bond to stabilize the native peptide into alpha helical structure, we get a peptide with higher cell permeability and stability compared to its parent form. Using gold nanorods (AuNRs) as delivery systems to deliver stable peptide into human MSCs, the delivery efficiency has been enhanced significantly by flow cytometry and cell fluorescent imaging. The intracellular delivery of stable peptide by AuNRs-PEI-based nanocarriers could inhibit the activation of LSD1, which together with hepatocyte growth factor (HGF) exhibits obviously synergistic effect to induce human MSCs differentiation. Furthermore, the hepatic marker genes AFP (alpha fetal protein) and ck19 are up-regulated by AuNRs-stable peptide (AuNRs- SP- PEI) with HGF. In conclusion, our study is the first time to use stable H3 peptide to inhibit LSD1 activation, which has been further delivered by AuNRs as nanocarriers into human MSCs.

## INTRODUCTION

As the largest organ in the human body, liver has strong abilities to regenerate and repair. Normally, the loss of liver tissue can be repaired by proliferation of the residual liver cells. However, chronic hepatitis virus, alcohol, trauma and other pathogenic factors will cause the abnormal proliferation of residual liver cells, disturb the regeneration of liver cells, resulting in hepatic failure and other hepatopathy [1–3]. Therefore, researchers devote lots of time to study liver regeneration and repair. During the last decade, cell-based therapies for hepatic repair and tissue engineering have been considered as the promising therapies for hepatopathy [4, 5]. Due to the ability of self-renewal and pluripotency, bone marrow-derived mesenchymal stem cells (BMSCs) have been confirmed as the incomparable representative for tissue regeneration and repair. Based on the previously reported studies, human BMSCs can be induced to differentiate into liver cells *in vitro*, which possess the morphological and functional characteristics of normal liver cells [6]. Therefore, the induction of human BMSCs differentiation into hepatocytes may provide a hopeful treatment of liver regeneration and repair. Until now, the normal methods are based on hepatocyte growth factors or drugs to induce human BMSCs differentiation, which take a long time and have side effects or toxicity. The development of new strategies is urgently needed for the treatment of liver diseases.

Lysine-specific demethylase 1 (LSD1) is the first identified histone demethylase, which specially removes mono- and dimethyl groups from methylated histone H3 at lysine 4 (H3K4) and lysine 9 (H3K9) [7, 8]. It is involved in DNA replication, transcription, and DNA repair. Recently, a great many reports have proved that LSD1 is indispensible for the pluripotency and proliferation of stem and cancer stem cells [9, 10], which is the new target for cancer therapies. Presently, much attention has attracted to LSD1 modulator exploiting. The LSD1 inhibitor researches major focus on small chemical molecules. For example, Zhang et al. has screened a series of small compounds which owned micromolar Kd value for LSD1. These molecules are usually highly unstable under physiological conditions. Moreover, the normal cell toxicity also a major problem pretends them go into clinical trial. Peptide based inhibitors, which possess many advantages compared with small molecules, have lower toxicity and higher binding affinity. While a main obstacle for peptide inhibitor is stability. Usually, a linear peptide will proteolysis fast when penetrate into cells where amount of enzymes existed. During the past decades, kinds of peptide stabilized strategies have been invented to constraint peptide into it native conformation of which binds to it partner. The disulfide bond strategy is the easiest accessible strategy compared with other strategies, which formed by two natural amino acids cysteine.

Nanotechnology is an emerging field that combine a wide range of technologies and has been successfully utilized in various fields [11–13], such as optics, manufacturing, materialogy and biomedicine, etc. Over the last decade, nanotechnology draws exponential increase of attentions in biomedical fields, particularly in the development of innovative methods as disease diagnosis tools and drug delivery systems [14]. To date, numerous nanoparticles have been employed in biomedical applications, including liposomes [15], carbon nanoparticles [16], quantum dots [17, 18], mesoporous silica nanoparticles (MSNs) [19] and gold nanoparticles [20, 21]. Gold nanorods have been considered as the “weapon” among these numerous nanomaterials, with controllable particle size, multiple surface functionalization, typical optical properties, high loading capacity and biocompatibility [22, 23].

In our study, we have designed LSD1 peptide inhibitors based on the crystal structure of LSD1 binding with histone H3. From the crystal complex of LSD1 and H3, we found the N-terminal nine amino acid peptide is the most important sequence to determine the binding. Recent published literatures also reported results that the N-terminal six amino acid segment is enough to inhibit LSD1. The Ala scan screening also proved that the N-terminal Pro and Arg is the hot spot for binding which form H-bonds and salt bridge with the LSD1 binding domain. In addition, the terminal Pro helps to cap alpha helical structure. Thus, we replaced the first and third amino acid in the C terminal and an oxidation reaction was performed. An i, i+3 positions disulfide bridge was formed similar to previous literatures. To assist stable peptides entering cells without degradation, we developed functionalized nanocarrier AuNRs to deliver stable peptides into human BMSCs. According to cell fluorescent imaging, stable peptides can be more effectively transported into human BMSCs by AuNRs. Furthermore, H3 stable peptides can sufficiently inhibit the activity of LSD1, which regulates the modifications of H3K4me1, H3K4me2 and H3K9me1 and the expression of stemness genes or differentiation genes. Importantly, stable peptides delivered by AuNRs contribute to the synergistic effect with hepatocyte growth factor (HGF), which effectively induced human BMSCs differentiation to hepatocytes. Compared to the single HGF treatment, AuNRs-stable peptides nanocomplex displays more significant effect on the efficient differentiation of human BMSCs to hepatocytes. We have designed and synthesized LSD1 stable peptide inhibitors. With AuNRs as drug delivery systems, LSD1 stable peptide inhibitors can be more efficiently transported into cells to initiate the differentiation of human BMSCs.

## RESULTS

### Protein-protein interactions are essential for all physiological process

**Figure 1:**
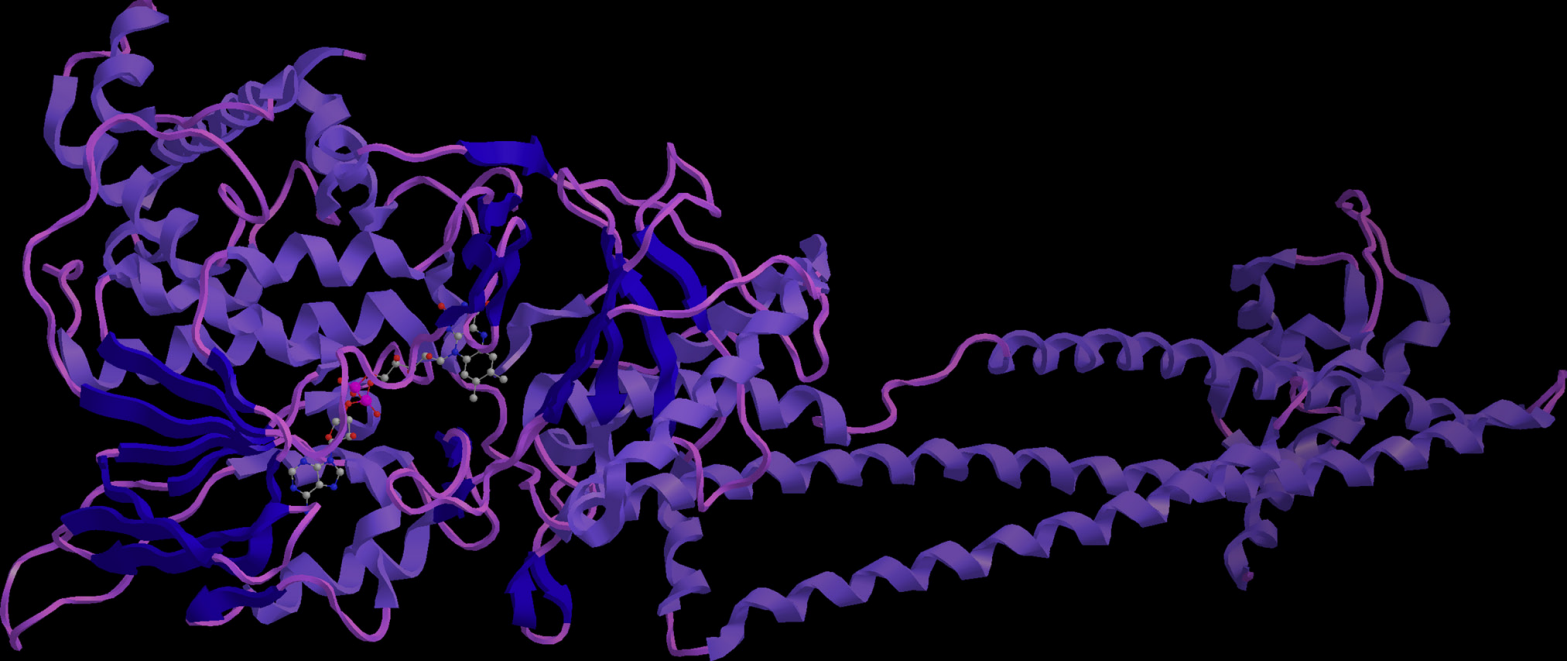
Schematic illustration of the crystal complex of LSD1 and histone H3 The interaction interface was consisted by alpha helix mediated protein-protein interactions.

Almost 60% PPIs are mediated by alpha helix. Design stabilized alpha helical structure to interfere the protein-protein interaction have received much attention during the past decades. LSD1 and histone H3 is a typical PPI, as LSD1 has very important biological function, and no stabilized peptide inhibitor has studied, design stabilized peptide to interrupt the LSD1 function is urgently needed. We carefully check the co-crystal structure of LSD1 and H3, we found the interaction interface is consisted by regular helical structure (Figure 1). Thus, we plan to introduce a C-terminal disulfide bridge to increase its proteolytic stability and cell permeability.

### The disulfide bond bridge is the most commonly structure motif in many native proteins, which is important to retain the secondary or three-dimension structure of proteins

**Figure 2:**
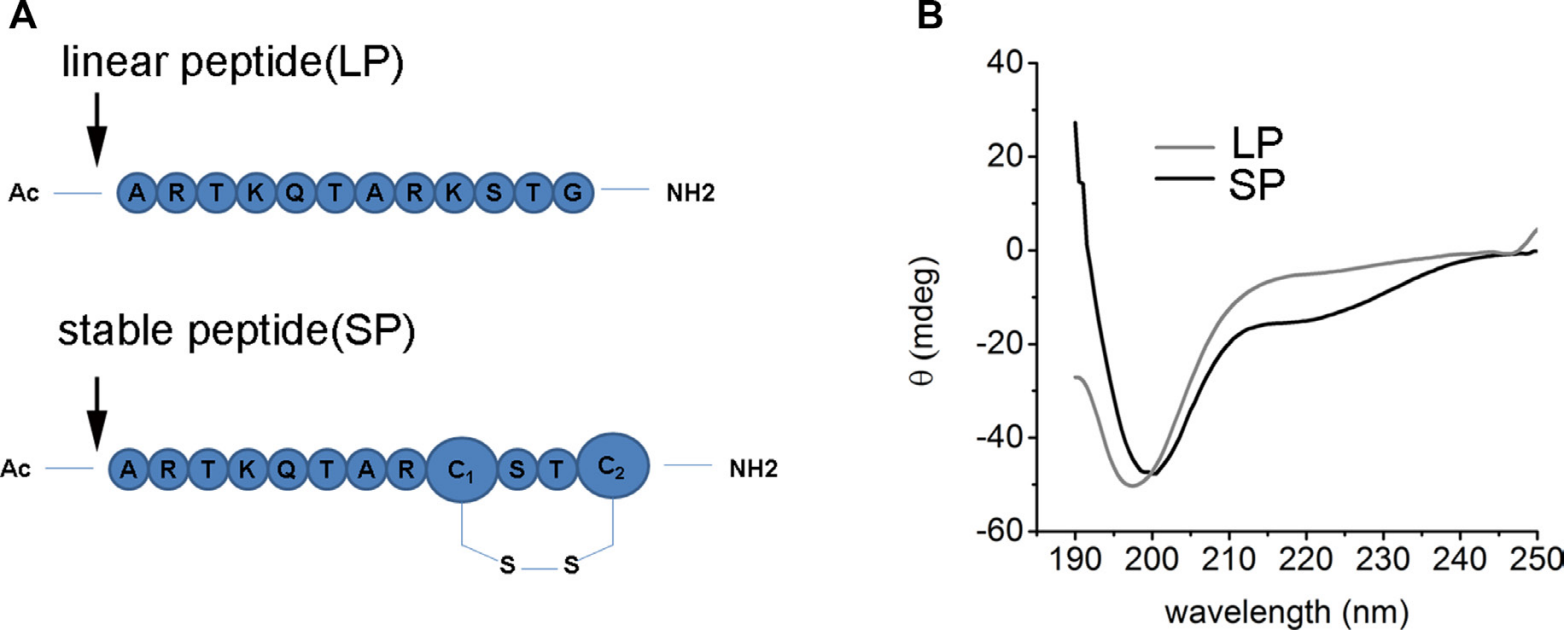
The characterization of LSD1 peptides (**A**) The sequence of LSD1 and LSD2 peptides. The disulfide bond was formed by the C terminal cysteine between the i, i+3 positions, (**B**) The CD spectra of LSD1/2 peptides in PBS buffer at 20^°^C. LSD1 is random coil while LSD2 peptide displayed some extent of alpha helicity.

Compared with other peptide stabilized strategies, disulfide bond is the easiest accessible and with littlest effect to the native structure thus will keep the native binding form. The LSD peptide in this article was based on the Fmoc-based solid phase peptide synthesis methodology (Figure 2A). In brief, MBHA resin with a load degree of 0.37 was used as solid support, then a 20% pyridine in DMF was used to deprotect the amino group protection group Fmoc, then 4eq of amino acid was mixed with a coupling reagent HCTU and base DIPEA to react with a bubbling N2 flux. This process was last until the last amino acid was coupling over and a capping reagent of acetic anhydride was used to cap the terminal amino group. After complete the peptide synthesis, the thiol protection group Trt was deprotected with a 3% TFA in DCM, the oxidation reaction of cysteine was performed based on the previous literature. Finally, the peptide was cleavage from the solid resin use a 95% TFA. Reverse-phase HPLC was used to separate the peptide, LC/MS was used to monitor the HPLC fraction. Then the peptide was lyophilized for other use. The circular dichroism spectra was used to characterize the secondary structure of LSD peptide (Figure 2B). For LSD1, it displayed no helicity in PBS buffer, while for LSD2, we can see two negative peak at 208 and 222 nm, it indicates a helical structure was formed. These results proved that the disulfide bond bridge helped stabilize peptide structure.

### Synthesis and characterization of MSNs

**Figure 3:**
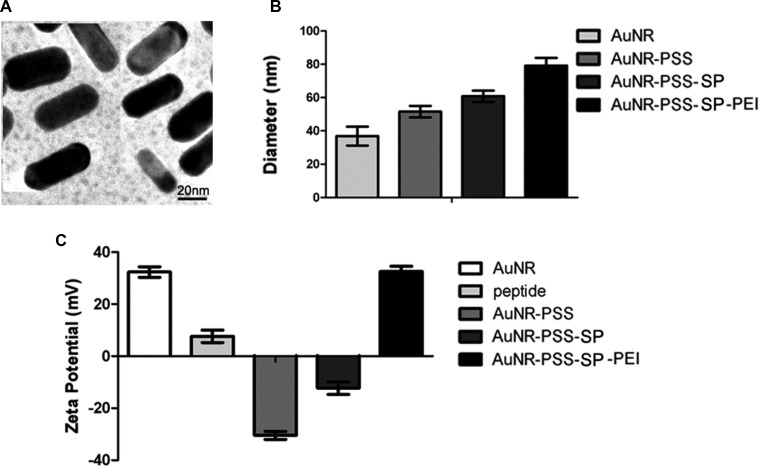
Morphology and characterization of gold nanorods (AuNRs) (**A**) The TEM image of AuNRs. The average diameter of the AuNRs is 38 ± 2.3 nm. Scale bars: 20 nm. (**B**) Diameter and (**C**) zeta potential of different AuNRs complex in ultrapure water measured by DLS.

Among numerous nanomaterials, AuNRs possess tunable particle size, unique optical properties and incomparable biocompatibility, which have been considered as the promising drug delivery system in biomedical field, especially cancer diagnosis and therapy. During the last decade, the study on AuNRs in biomedical applications exhibits a tremendous increase, with well-developed methods to control the structure as well as their size. In our current study, AuNRs were made by two-step seed-mediated growth method adapted from previously established procedures [24, 25]. By transmission electron microscopy, we found that the AuNRs appeared uniform in particle size and were also in highly dispersible spherical shape in ultrapure water (Figure 3A). Hydrodynamic size distribution of the AuNRs was then analyzed by dynamic light scattering (DLS) technique (Figure 3B). The bared AuNRs displayed a size of 38 ± 2.3 nm. The particle size had been changed among different AuNRs nanocomplex. During the typical synthesis, CTAB was used as surfactant, which could form AuNRs wall around the micelles of the surfactant, and give the AuNRs positive charge (+33.5 mV) (Figure 3C). To load the peptides which have positive charge (+9.1 mV), the negative polymer PSS was functionalized into AuNRs, which exhibited negative charge (−31.8 mV). As shown in Figure 3C, the zeta potential of AuNRs-stable peptide remained the negative charge (−13.3 mV). Due to the negative charge on the cell membrane, the positive polymer PEI was utilized to change the charge of the AuNRs-SP-PEI nanocomplex (+ 32.1 mV), which showed strong electrostatic interaction with plasma membrane of the cells in our following experiments.

### *In vitro* cellular delivery of AuNRs-stable peptide nanocomplex in human BMSCs

**Figure 4:**
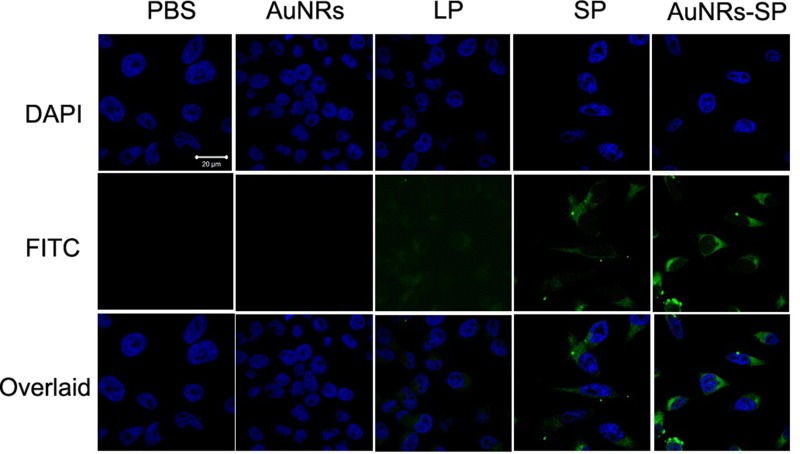
Fluorescent images of human BMSCs Human BMSCs were treated with PBS (as blank), AuNRs, linear peptide (LP), stable peptide (SP) and AuNRs-SP for 4 hours. Peptides are modified with FITC and the signals from peptide-FITC in cells are assigned in green. DAPI is used to label nuclear DNA and are assigned in blue. Scale bars: 20 μm.

**Figure 5:**
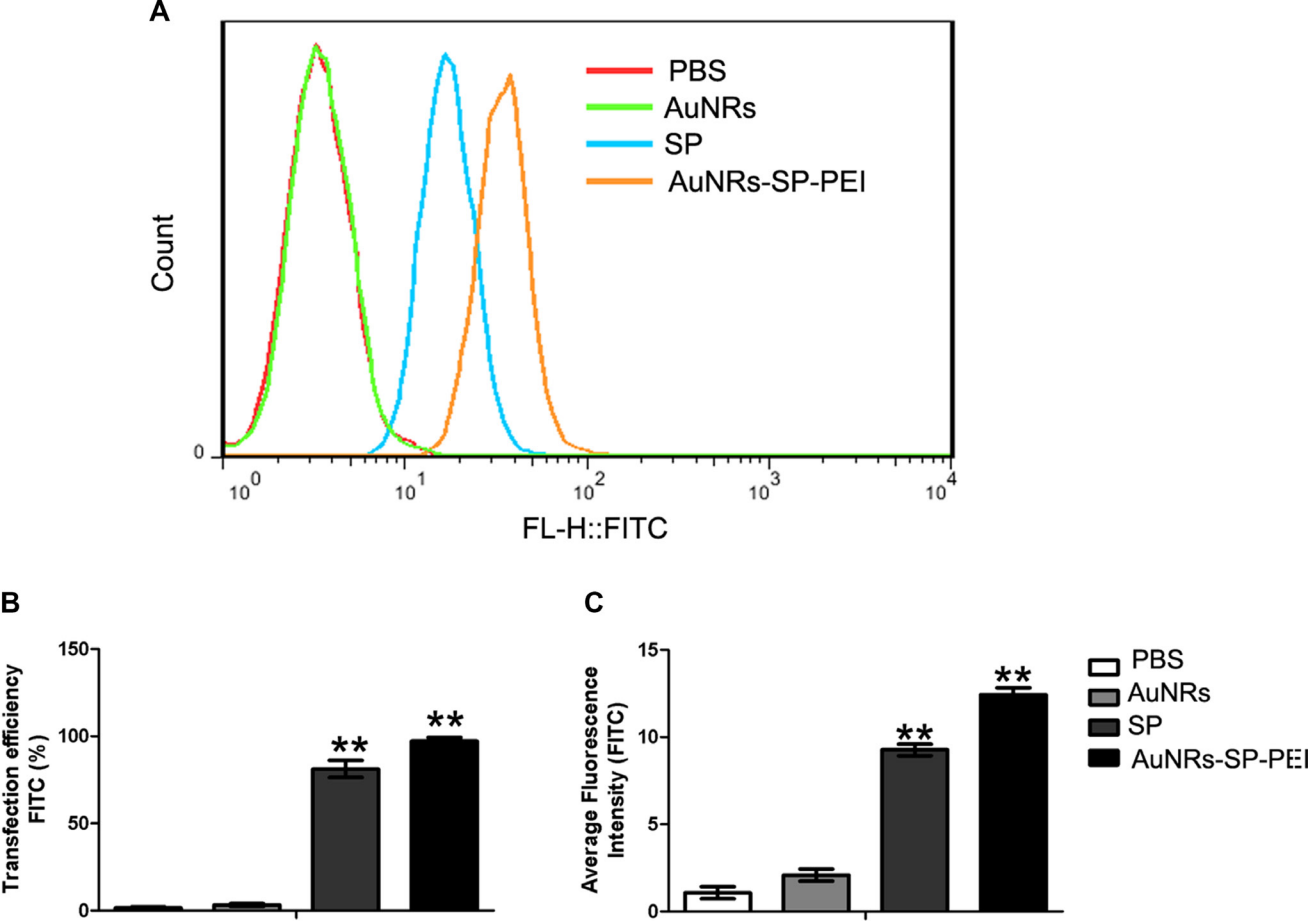
Delivery efficiency of human MSCs evaluated by flow cytometry (**A**) Human mesenchymal stem cells were treated with PBS (as blank), AuNRs, stable peptide (SP) and AuNRs-SP for 4 hours and the statistical count of cells were gathered by flow cytometry. (**B**) The delivery efficiency and (**C**) average fluorescent intensity of each group respectively. Average fluorescence intensity presents the FITC intensity in the cells. The delivery efficiency is defined as the ratio between delivered cell counts to total cell counts. Data were presented as the means ± SEM of three independent experiments. **p < 0.01 vs control.

Cell permeability is the main hurdle of peptide based drugs. There are many factors affecting peptide's permeability including its conformation. Increasing a stabilized peptide helical content will improve it permeability property. We wish a disulfide bridge constraint peptide will increase the stability and thus increase it permeability of peptide. In order to detect the cell permeability of LSD2 after cyclization, we connect a FITC fluorescence tag to the N terminal of the peptide. Aimed to decrease the influence of FITC to the peptide, a beta alanine was used to separate of the peptide and FITC. The confocal microscope and flow cytometry were used to measure the cellular uptake of our peptides. Both the confocal and FACS indicated that the LSD2 peptides have higher cell permeability than LSD1 (Figure 4 and Figure 5). Nowadays, targeted delivery drugs have been a very hot field. The targeted delivery will greatly improve the drug efficiency and decrease side effect. Here we utilized the AuNRs as the carrier to deliver the LSD peptide to the human MSCs. After binding to the AuNRs, the cellular fluorescence intensity was significantly increase (Figure 4 and Figure 5).

### Delivery efficiency of AuNRs nanocomplex into human MSCs evaluated by flow cytometry

In order to estimate the corresponding delivery efficiency and average fluorescent intensity of AuNRs nanocomplex, human BMSCs were treated with PBS as control, AuNRs, stable peptide (SP) and AuNRs-SP for 4 hours and then were analyzed by flow cytometry. Figure 5A shows statistical count of the FAM intensity in human MSCs treated with different AuNRs nanocomplexes. Based on the intensity of the fluorescent signals, cells treated with AuNRs-SP-PEI exhibited the stronger signals than the signals from cells treated with SP alone. Cells treated with PBS and AuNRs were considered as negative control. The delivery efficiency and average fluorescent intensity were analyzed by Flowjo software. The strongest delivery efficiency (98.55 ± 0.20%) and average fluorescent intensity were detected from cells treated with AuNRs-SP-PEI. Cells treated with SP alone showed weaker delivery efficiency (85.55 ± 2.50%) and average fluorescent intensity. We didn't observe any fluorescent signals from the cells treated with either PBS or AuNRs. Therefore, the data were in consistency with the cell fluorescent imaging results, which indicated AuNRs were the excellent drug delivery system to assist peptide entrance into cells.

### Inhibition of LSD1 activity has synergistic effect with HGF to differentiate human BMSCs into hepatocytes

**Figure 6:**
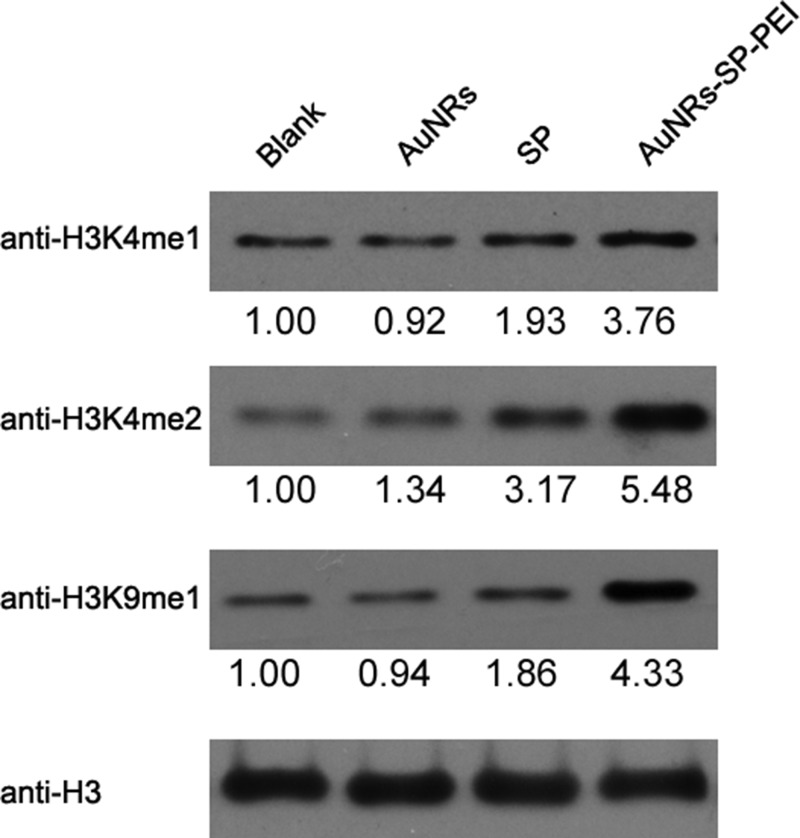
Inactivation of LSD1 causes similar changes of histone methylation in human BMSCs Human BMSCs were treated with PBS, AuNRs, stable peptide alone and AuNRs-SP for 24 h, and the methylation of histones H3 were monitored by Western blotting, histone H3 as a loading control. Quantification for each lane was shown below, normalized by loading control.

Lysine-specific demethylase 1(LSD1) is the first identified histone demethylase, which can change the epigenetic modification of histone by removing mono- and dimethyl groups from methylated histone H3 at lysine4 (H3K4) and at lysine 9 (H3K9) [9, 26, 27]. LSD1 is critical for the pluripotency in stem and cancer stem cells, which has been considered as the novel target for cancer therapy and other diseases. Therefore, to explore whether the stable peptide can inhibit the activity of LSD1, we examined and compared the changes of epigenetic methylation of histones H3K4 and H3K9 in human MSCs (Figure 6). Human BMSCs were treated with PBS as control, AuNRs, stable peptide (SP) alone and AuNRs-SP nanocomplex respectively for 48 hours and then the changes of histones H3K4 and H3K9 methylation were analysed by Western Blotting (Figure 6). Compared with the PBS-treated and AuNRs-treated groups, inactivation of LSD1 by stable peptide or AuNRs-SP nanocomplex induced the accumulation of mono- and di-methylated H3K4 and H3K9. The accumulation in AuNRs-SP treated group was more apparent than the stable peptide-treated group. Therefore, the AuNRs-SP nanocomplex is superior to inhibit the activity of LSD1.

**Figure 7:**
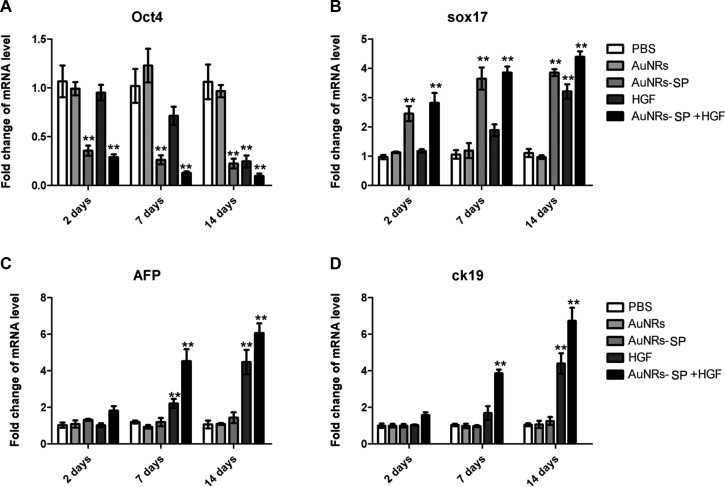
Stable H3 peptide delivered by AuNRs in human BMSCs repressed the mRNA expressions of downstream genes Human BMSCs were treated with PBS (as blank), AuNRs, hepatocyte growth factor (HGF), AuNRs-SP and AuNRs-SP+HGF for 14 days and the mRNA levels of downstream genes (**A**) Oct4, (**B**) Sox17, (**C**) AFP (alpha fetal protein) and (**D**) ck19 were analyzed by RT-PCR. Data were presented by the mean ± SEM of three independent experiments. **p < 0.01 vs control.

After the demonstration of inactivation of LSD1 by AuNRs-SP nanocomplex, we further explored the directional differentiation of human BMSCs into hepatocytes after the combined treatments of HGF and AuNRs-SP nanocomplex. The human BMSCs were treated with PBS as control, AuNRs, AuNRs-SP nanocomplex, HGF alone and AuNRs-SP nanocomplex+HGF respectively for 2 days, 7 days and 14 days (Figure 7). Consequently, the expression of stemness gene Oct4, differentiation gene Sox17 and two hepatic marker genes, AFP and ck19 in the human MSCs were analyzed by RT-PCR. Comparing with PBS-treated group and AuNRs-treated group, human MSCs were induced to differentiate by AuNRs-SP nanocomplex with the decreased Oct4 and increased Sox17 (Figure 7A and 7B). Under the combined treatment of AuNRs-SP nanocomplex and HGF, the two hepatic marker genes AFP and ck19 were significantly up-regulated at mRNA level during 7 days (Figure 7C and 7D). It was worth noting that HGF alone could not change the expression of AFP and ck19 until 14 days, which indicates the synergistic effect of AuNRs-SP nanocomplex and HGF to induce human MSCs differentiation into hepatocyte. Together, the time of differentiation into hepatocyte from human BMSCs, treated with the both AuNRs-SP nanocomplex and HGF, could be shortened from 14 days to 7 days.

## DISCUSSION

In this study we designed a novel LSD1 inhibitor based on it binding partner H3 protein. Based on the crystal complex, we knew that the interaction interface was consisted by alpha helix mediated protein-protein interactions. Though there are many small molecule inhibitor of LSD1 during the past decades, their toxicity and intracellular instability made them improper for clinical trial. Here we report the first stabilized peptide inhibitor for LSD1, which is more specific to the LSD1 protein, low toxicity and higher cell permeability.

Moreover, disulfide bond strategy for peptide stabilization has many advantages, First, it does not need unnatural amino acid, and it is fast accessible; second, there are many disulfide bridges inner native proteins, thus our stable peptide has little immunogenicity; third, a stable structure helps the peptide bind more tightly to the target protein and it has little interference to the native peptide structure thus remain it bonding mode.

Targeted therapy has made great success in these years. Compared to the non-targeted therapy, it has higher drug efficiency. In addition, the nanomaterial as carrier will further reinforce the cellular uptake. The gold nanorods is a bar of gold nanoparticles, it has good biological compatibility, low toxicity, high stability, and chemical stability, as well as strong X-ray absorption, thus it can be applied to all parts of the body. The tumor cells have strong absorption properties of gold nanorods, thus through static vein injection, makes the enrichment of gold nanorods in tumor tissues. In our study, the AuNRs significantly increase the cellular uptake of our peptides. The mRNA analysis also indicated that AuNRs-peptide combination has the most apparent effect to the oct4 and sox 17.

Nowadays, the therapy of liver disease major focused on the small chemical molecules or Chinese herbal medicine. The eradicate of liver disease is very hard. Our stable peptide is the first research in this field. As liver disease is very hard to cure, new modality of drug forms is urgently needed and our research provides new possibility for developing liver disease therapy.

## MATERIALS AND METHODS

### Chemicals and reagents

All reagents including amino acids and resins were purchased from GL Biochem (Shanghai), Shanghai Hanhong Chemical Co., J&K Scientific or Energy Chemical and were used without further purifications. Hexadecyltrimethylammonium bromide (CTAB, > 98.0%), sodium 3-methylsalicylate (> 97.0%), 5-bromosalicylic acid (> 98.0%), 5-aminosalicylic acid (> 98.0%), sodium salicylate (99%), sodium borohydride (NaBH_4_, 99%) were purchased from Alfar. Hydrogen tetrachloroaurate trihydrate (HAuCl_4_·3H_2_O) was purchased from Acros Organics. L-Ascorbic acid (BioUltra, g99.5%), silver nitrate (AgNO_3_, > 99%) and hydrochloric acid (HCl, 37 wt % in water) were purchased from Sigma Aldrich. Ultrapure water produced with a Milli-Q Integral 5 system was used in all experiments. All glassware was cleaned with aqua regia, rinsed extensively with water, and dried before use.

### Peptide synthesis

A 10 mL peptide synthesis flask was filled with 100 mg of Rink amide resin. The resin was washed with CH_2_Cl_2_ (3 × 1 mL) and DMF (3 × 1 mL). Piperidine (20% in DMF, 2 mL) was added to the reaction flask, which was then agitated for 30 min. After draining the piperidine solution, the resin was washed with DMF, CH_2_Cl_2_, MeOH, CH_2_Cl_2_ and DMF (3 × 1 mL each). Fmoc-protected amino acids (4 equiv, 0.15 mmoL) in 1 mL DMF was added to the reaction flask with HCTU (4 equiv, 0.15 mmoL) and DIEA (8 equiv, 0.3 mmoL), which was agitated for 2 h. The resin was washed again with DMF, CH_2_Cl_2_, MeOH, CH_2_Cl_2_ and DMF (3 × 1 mL each). This procedure was repeated until all amino acids were coupled to the resin. The oxidation of disulfide bond was performed referred to previous literature. In brief, the synthesized peptide was cleavaged from the resin, and thenoxidized with excess iodine in 1:1 trifluoro-ethanol/water, and the products were isolated by HPLC.

### Circular dichroism

LSD1/2 were dissolved in potassium phosphate solution (pH 7.0) to concentrations of 10–100 μM. The spectra were obtained on an Applied Photophysics Chirascan Circular Dichroism Spectrometer at temperature of 20°C using the following standard measurement parameters: wavelength, 190–250 nm; step resolution, 0.2 nm; speed, 20 nm/sec; accumulations, 10; response, 1 sec; bandwidth, 1 nm; path 3 length, 0.1 cm. Every sample was scanned twice and the final CD spectra was averaged and smoothed using the Pro-data Viewer.

### Fluorescence imaging

LSD peptide or AuNRs complex were first dissolved in DMSO to make a 1 mM stock and then added to cells to a final concentration of 5 μM. The cells were incubated with peptides for 1 hour at 37°C. After incubation, cells were washed 3 times with PBS and then fixed with 4% formaldehyde (Alfa Aesar, MA) in PBS for 10 minutes. They were then washed 3 times with PBS and stained with 1 μg/mL 4′, 6-diamidino-2-phenylindole (DAPI) (Invitrogen, CA) in PBS for 5 minutes. Images of peptide localization in cells were taken on PerkinElmer confocal microscopy. Image processing was done using Volocity software package (Zeiss Imaging, Germany).

### Synthesis of AuNRs

AuNRs were prepared via the previously reported two-step seed-mediated growth method with modifications [24, 25]. Firstly, 500 μL of ice-cold NaBH_4_ solution (10 mM) was quickly added to 200 μL of 0.01 M HAuCl_4_ dissolved in 0.1 M CTAB solution, and stirred for 5 min. for the reaction continued for 2 h to form the CTAB-capped Au nanoparticles as seed solution. 95 mL of CTAB solution (0.1 M), 950 μL of AgNO_3_ solution (0.01 M) and 5 mL of HAuCl_4_ solution (0.01 M) were mixed together to synthesize the growth solution. Subsequently, 550 μL of freshly prepared L-ascorbic acid solution (0.1 M) was added to the above mixture as a weak reducing agent. After swirling for 10 min, 100 μL of CTAB-capped Au nanoparticle seeds were added and the solution was left overnight. The resulting AuNRs were separated from the reaction solution by centrifugation at 10,000 rpm for 20 min, and washed with deionized water three times to remove any residual reactants.

### AuNRs characterizations

Hydrodynamic size distribution profile, as well as the zeta potential of the AuNRs were assessed by a particle size analyzer system (90 Plus, Brookhaven Instruments). AuNRs solution was dispersed drop by drop onto a carbon coated 300 mesh copper grid (Carbon Type-B, Ted Pella, Inc.). After samples were coated on the grid, 10 μL 2% uranyl acetate solution was added drop by drop on the grid for negative staining. Transmission electron microscopic images were acquired using a JEM-2010 transmission electron microscope with 200 KV acceleration voltage.

### Loading peptides by AuNRs

Due to the positive charge of peptides, the synthesis AuNRs with CTAB which has positive charge should be changed for electrostatic interaction with peptides. First, AuNRs was centrifuged at 10,000 rpm for 20 min and the AuNRs precipitate was redispersed in ultrapure DI water. Subsequently, the resulting AuNRs mixture was added slowly to 5% PSS under vigorous stirring 12 hours, and then removed the unbound PSS by centrifugation (10,000 rpm, 20 min). The AuNRs-PSS (100 pM) and peptide-FITC (100 μg) were added in 0.1 mL of ultrapure water, and subsequently stirred for 24 h. The mixture was then centrifuged at 10,000 rpm for 20 min to discard the supernatant. The absorption of the original solution and the supernatant were compared to determine the amount of peptide-FITC bound to AuNRs using UV/Vis spectroscopy. To enhance the positive charge of AuNRs-peptide, PEI (poly-ethylene imine) was used to interact with AuNRs-peptide to form AuNRs-peptide-PEI.

### Cell culture

The human bMSCs PCS-500-012^TM^ were purchased from American Type Culture Collection and verified on arrival, which were then cultured in BMSCs basal medium (Hyclone, USA) containing 7% fetal bovine serum, 0.25 μg/mL amphotericin B, 15 ng/mL rh IGF-1, 125 pg/mL Rh FGF-b, 10 μg/mL gentamicin, 2.4 mM L-alanyl-L-glutamine, 100 μg/mL penicillin and 100 μg/mL streptomycin. Cells were cultured at 37°C in a humidified incubator with 5% CO_2_.

### Flow cytometry

To test the delivery efficiency of AuNRs, human BMSCs were cultured in the 6-well plates and were treated with with PBS, AuNRs, stable peptide (SP) and AuNRs-SP separately for 4 h. The cells were then washed twice with PBS and harvested after trypsinization, followed by centrifugation at 1,000 rpm for 5 min to remove the trypsin. The cells were then analyzed using FACSCalibur (Becton Dickinson, Mississauga, CA, USA). The delivery efficiency and average fluorescent intensity were determined by the use of Flowjo software.

### RNA isolation and quantitative RT-PCR

After human BMSCs were treated with PBS, AuNRs, HGF, AuNRs-SP and AuNRs-SP+HGF for 14 days, total RNA was extracted using TRIzol (Invitrogen, Carlsbad, CA, USA) and was quantitated by Nano Drop 2000. 2 μg total RNA was reverse transcribed to cDNA using a reverse transcriptase kit (Takara, Japan) according to the manufacturer's instructions. The mRNA levels of the target genes were quantified by RT-PCR, using GAPDH as normalization. Primers used in this study: Oct4 forward 5′-GAC AAC AAT GAG AAC CTT CAG-3′ and reverse 5′-CTG GCG CCG GTT ACA GAA CCA-3′; Sox17 forward 5′-GAG CCA AGG GCG AGT CCC GTA-3′ and reverse 5′-CCT TCC ACG ACT TGC CCA GCA-3′; AFP forward 5′-AGC GAG GAG AAA TGG TCC GG-3′ and reverse 5′-GGA CAT CTT CAC CAT GTG-3′; Ck19 forward 5′-GCA CTA CAG CCA CTA CTA CAC-3′ and reverse 5′-CTC ATG CGC AGA GCC TGT-3′.

### Statistical analysis

Data were presented as the mean ± SEM of triplicate experiments. All statistical analysis was performed using SPSS 11.0. Student's unpaired two-tailed *t* test was used to compare difference of two groups. Statistical significance is accepted at *p* ≤ 0.01, which represents very significant differences.

## CONCLUSIONS

In summary, we have designed LSD1 peptide inhibitors based on the crystal structure of LSD1 binding with histone H3 and stabilized the peptide by disulfide bond strategy. With the delivery by functionalized AuNRs, LSD1 stable peptide inhibitors are sufficiently transported into human BMSCs and have induced the differentiation of human BMSCs into hepatocytes. The successful delivery could be observed by fluorescent microscope and flow cytometry. Western blot assay also has proved LSD1 stable peptide inhibitors could inhibit the activity of LSD1. It is worth noting that the combination of HGF and AuNRs-stable peptides nanocomplex could shorten the time that human BMSCs differentiate towards hepatocytes, with the up-regulation of hepatic marker genes AFP and ck19. Based on these results, the combination of nanotechnology and peptide engineering will be the promising strategies for clinical applications in biomedical fields.
